# *Mycobacterium leprae* Hsp65 administration reduces the lifespan of aged high antibody producer mice

**DOI:** 10.1186/1742-4933-11-6

**Published:** 2014-03-26

**Authors:** Estevam José Baldon, Eliana Blini Marengo, Marcelo de Franco, Nancy Starobinas, Valquiria Bueno, Osvaldo Augusto Sant’Anna

**Affiliations:** 1 Laboratório de Imunoquímica, Instituto Butantan, Avenida Vital Brazil 1500, 05530-900 São Paulo, Brasil; 2 Hospital Israelita Albert Einstein, Avenida Albert Einstein, 627/701, 05652-000 São Paul, Brasil; 3 Laboratório de Imunogenética, Instituto Butantan, Avenida Vital Brazil 1500, 05530-900 São Paulo, Brasil; 4 Departamento de Microbiologia, Imunologia e Parasitologia, UNIFESP, Rua Botucatu 862, 04023-062 São Paulo, Brasil

**Keywords:** Heat shock protein, Hsp65, Aging, Immunosenescence, Antibody response

## Abstract

**Background:**

Aging process may result in immune modifications that lead to disruption of innate and acquired immunity mechanisms that may induce chronic-degenerative events. The heat shock proteins (Hsp), phylogeneticaly conserved among organisms, present as main function the ability of folding and refolding proteins, but they also are associated with chronic-degenerative disorders. Here were evaluated the role of *M. leprae* native Hsp65 (WT) and its point-mutated (K^409^A) on survival and anti-DNA and anti-Hsp65 antibody production of aged genetically selected mice for high (H_III_) and low (L_III_) antibody production; data from 120- and 270-days old mice (named “adult” or “aged”, respectively) were compared.

**Results:**

WT Hsp65 administration induces reduction in the mean survival time of adult and aged female H_III_ mice, this effect being stronger in aged individuals. Surprisingly, the native protein administration increased the survival of aged female L_III_ when compared to K^409^A and control groups. No survival differences were observed in aged male mice after Hsp65 proteins inoculation. We observed increase in IgG1 anti-Hsp65 in WT and K^409^A aged H_III_ female mice groups and no marked changes in the anti-DNA (adult and aged H_III_) and anti-Hsp65 IgG1 or IgG2a isotypes production in adult H_III_ female and aged male mice. L_III_ male mice presented increased anti-DNA and anti-Hsp65 IgG2a isotype production after WT or K^409^A injection, and L_III_ female groups showed no alterations.

**Conclusions:**

The results revealed that the WT Hsp65 interferes with survival of aged H_III_ female mice without involvement of a remarkable IgG1 and IgG2a anti-DNA and anti-Hsp65 antibodies production. The deleterious effects of Hsp65 on survival time in aged H_III_ female mice could be linked to a gender-effect and are in agreement with those previously reported in lupus-prone mice.

## Background

Aging is defined as progressive alterations of biological functions, leading to the onset of diseases and reduced ability to respond to external stimuli [1]. Alongside with the physiological aging events, the immunosenescence accumulates potential modifications in immunological functions and its components. The most important changes include the decrease of the absolute number of lymphocytes, alterations of the activation status of T cells, increasing of serum levels of immunoglobulin (mainly IgA and IgG), limitation of the protective response of specific high affinity antibody, amplification of autoantibody production and a switch for a Th2 pattern of cytokine response [2]. The altered processes in advanced age also result in the failure of self and non-self discrimination [3] and disruption of the innate and acquired immunity mechanisms, which may result in chronic-degenerative events and subsequent loss of life quality [4-7]. Altogether, these modifications lead to an increased vulnerability to infections [8,9], reduced response to vaccines [10], development of tumors [11,12], and autoimmune or inflammatory diseases [13,14]. In addition, disorders related to the abnormal processing, modification, and aggregation of proteins typically linked to biological properties of the heat shock proteins (Hsp) are reported [15,16].

Drastic alterations in physiological responses to stressful events are related to Hsp production [17-19]. Hsp are phylogeneticaly conserved molecules among evolutionary scale [20,21] which assist misfolding molecules and control the arising of toxic protein aggregates, supporting the folding and unfolding of polypeptides for degradation by proteolytic machinery [22,23]. Hsp65, the most abundant and immunogenic protein of mycobacteria [24], is considered a toxin and dominant antigen in infectious diseases, capable of induce humoral and cellular immune responses [25-27]. Reports evidenced the immunodominant role of the Hsp60 family in infectious processes [28], besides of the role played in inflammatory processes such as arthritis, type I diabetes, multiple sclerosis and atherosclerosis [29-32]. In the opposite, some studies demonstrate its regulatory function on immune suppression in rheumatoid arthritis [33] and type I diabetes [34].

Previously, our group evaluated the immunomodulatory effects *in vivo* of *M. leprae* Hsp65 on genetically homogeneous (NZBxNZW)F_1_ hybrid female mice that develop systemic lupus erythematosus (SLE); the results showed that the native protein (WT) aggravates the lupus progression in mice [35]. On the other hand, the K^409^A, a point-mutated Hsp65 [36], revealed a potential in mitigating lupus aggravation in these mice [37]. Hsp65 administration also increased eye lesions in mice susceptible to the development of autoimmune uveitis [38].

Autoimmune diseases are more frequent in aged and in female individuals [39] and thus we asked whether Hsp65 interference in autoimmunity is age and/or gender-related. Reports of Hsp65 interference in autoimmunity and other biological alterations occurring during the immunosenescence process are related to gender and aging [40]. These findings lead us to investigate whether *M. leprae* Hsp65 is also involved in alterations of aged individuals, as the immunosenescence process can lead to the onset of autoimmunity. It was assessed the role played by passive administrations of WT and mutant K^409^A Hsp65 on the lifespan and antibody production of aged H_III_ and L_III_ mice. We conclude that the WT protein administration interferes with the survival of aged and adults H_III_ female mice, even though the anti-DNA and anti-Hsp65 antibody production was not markedly changed. As no significant changes in male mice survival and antibodies production were observed we conclude that Hsp65 effects were gender-related.

## Results

### WT Hsp65 administration reduces the lifespan of H_III_ female mice

**Figure 1:**
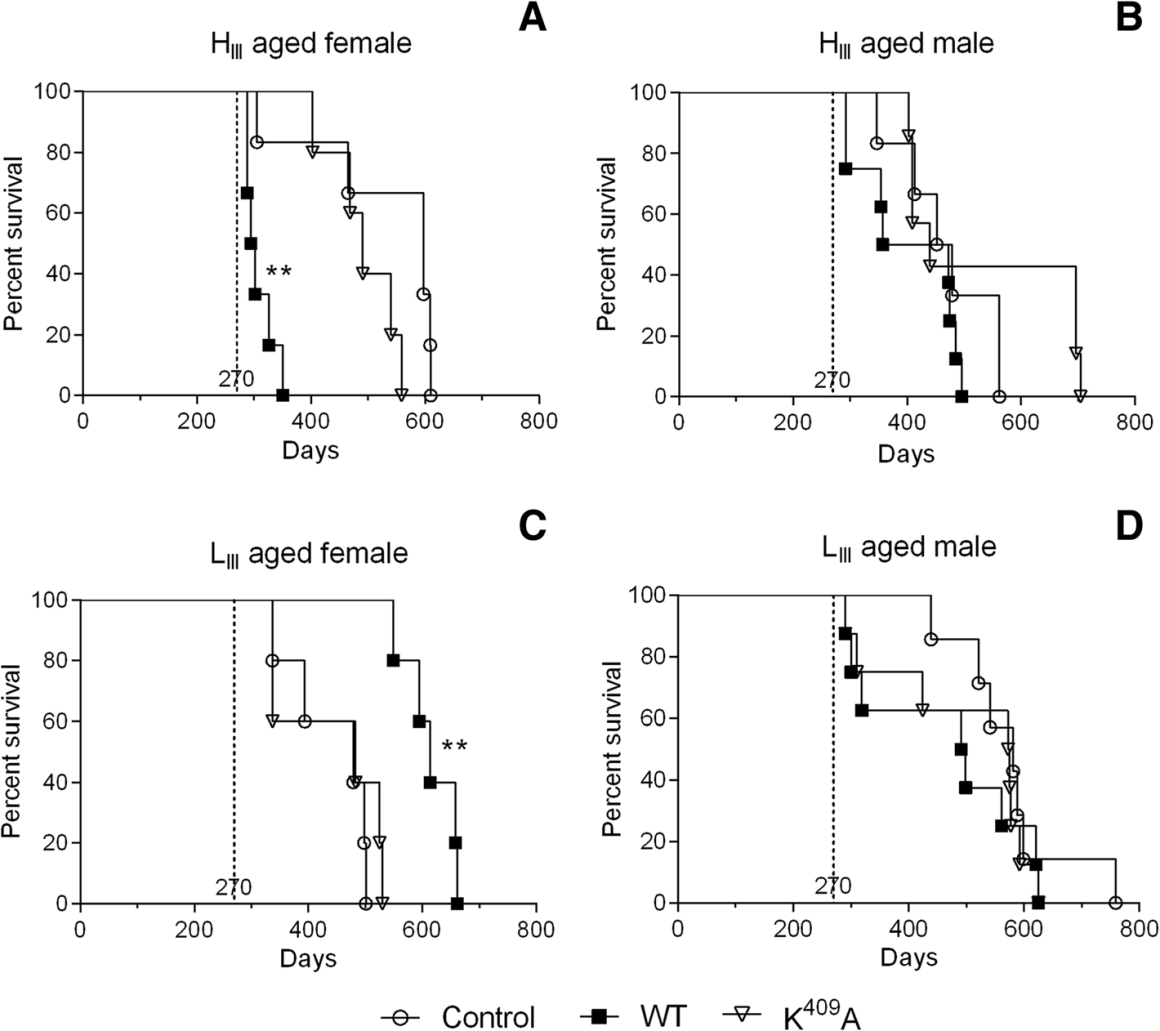
**Mean survival time of aged mice inoculated with Hsp65 proteins.** Female H_III_**(A)** and L_III_**(B)**, and male H_III_**(C)** and L_III_**(D)** animals were intraperitoneally injected with 2.5 μg/animal of WT or K^409^A with 270-days old (dotted line); whereas control mice received PBS. **p < 0.01 (log rank test Mantel-Cox). The control group was used as reference for statistical analysis.

Male and female two hundred-seventy-days old (aged) mice were inoculated intraperitonially with a single dose of 2.5 μg/animal of WT or K^409^A Hsp65 in 0.2 mL of PBS, or only PBS as control group. Mice were observed until death for mean survival time (MST) and environmental variance (V_E_) determination (Table 1). Figure 1A illustrates the survival reduction of aged H_III_ female mice inoculated with the native protein (308 ± 25 days, *p <* 0.01) compared to controls (530 ± 123 days). Also it was observed a decrease of 24-fold in the WT group phenotypic variance (V_E_ = 635 days in WT group versus V_E_ = 15293 days in control group). Conversely, Figure 1B shows that aged L_III_ females inoculated with WT Hsp65 presented higher MST (615 ± 46 days; *p <* 0.01) compared to K^409^A (442 ± 97 days) and control group (441 ± 72 days) and a decrease in V_E_ (2180 days) in contrast with control (5328 days) and K^409^A (9590 days) groups. No survival differences were observed in aged male mice from both H_III_ and L_III_ in all experimental groups (Figure 1C and D).

**Table 1:** **Mean survival time (days) and environmental variance of H**_**III **_**and L**_**III **_**mice**

		**♀ H**_**III **_**adult**	**♀ H**_**III **_**aged**	**♀ L**_**III **_**aged**	**♂ H**_**III **_**aged**	**♂ L**_**III **_**aged**
**Control**	MST	614 ± 177	531 ± 123	442 ± 72	469 ± 84	575 ± 97
	V_E_	31473	15294	5329	7141	9534
		(n = 4)	(n = 6)	(n = 5)	(n = 6)	(n = 7)
**WT**	MST	466 ± 134*	308 ± 25**	615 ± 46**	403 ± 88	463 ± 141
	V_E_	18182	635	2180	7802	20018
		(n = 4)	(n = 6)	(n = 5)	(n = 8)	(n = 8)
**K**^**409**^**A**	MST	665 ± 37	492 ± 61	442 ± 97	537 ± 151	496 ± 134
	V_E_	1422	3823	9591	23097	18093
		(n = 4)	(n = 5)	(n = 5)	(n = 7)	(n = 8)

Mean survival time (MST) and environmental variance (V_E_) are shown for control, WT (native Hsp65) and K^409^A (mutant K^409^A Hsp65) injected mice groups, males (♂) and females (♀). The values are displayed as plus/minus standard deviation. The number of subject used for each treatment is indicated in parenthesis. *p < 0.05 and **p < 0.01 related to the control of each mice group.

**Figure 2:**
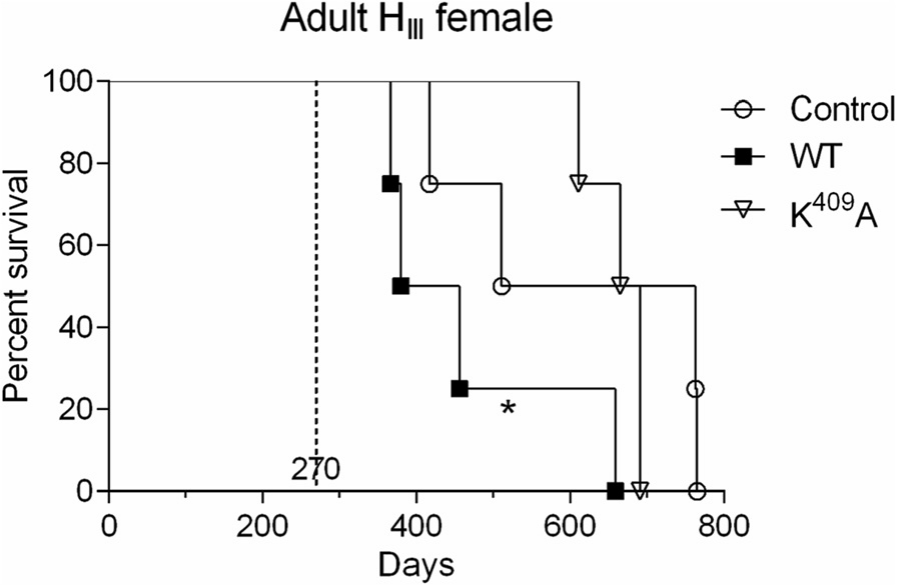
**WT Hsp65 protein influences on survival of adult H**_**III **_**female mice.** Female adults mice were intraperitoneally inoculated with 2.5 μg/animal of WT, K^409^A proteins, or PBS (control group) at 120-days old (dotted line). *p < 0.05 (log rank test Mantel-Cox). The control group was used as reference for the statistical analysis.

Since the lifespan of aged H_III_ females (270-days old) was shortened after WT Hsp65 inoculation, we asked whether the same effect could be observed in adult H_III_ female mice (120-days old) injected with Hsp65 (Figure 2). Adult H_III_ female mice showed a MST reduction after WT protein administration (466 ± 134 days; *p <* 0.01) when compared to K^409^A injected group (665 ± 37 days). The first death occurred 247 days after inoculation of the native molecule, and the environmental variance was lower in mutant- (1422 days, 22-fold less) and WT-inoculated animals (18181 days, about 2-fold less) compared to control group (31473 days).

It is noteworthy that in mice from both lines and ages no weight loss, piloerection or ascites were detected.

### Anti-DNA and anti-Hsp65 antibody production are altered after WT Hsp65 injection

**Figure 3:**
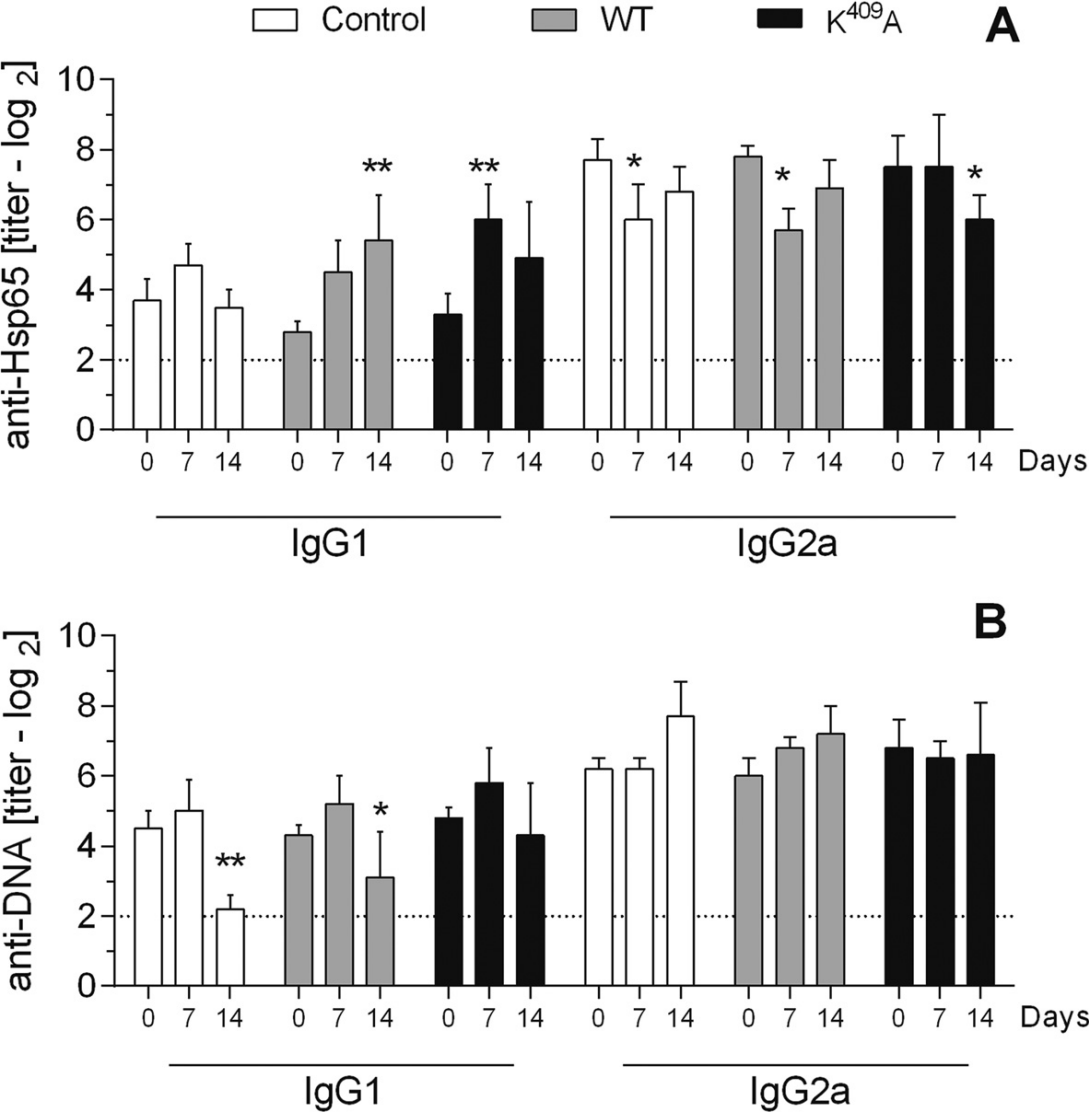
**Anti-Hsp65 and anti-DNA antibodies production in aged H**_**III **_**female mice.** Anti-Hsp65 IgG1 and IgG2a **(A)**, and anti-DNA IgG1 and IgG2a **(B)** isotype production in the serum of aged mice (3–6 animals/group) inoculated with WT, K^409^A Hsp65, or PBS (control). Antibody titers were set by ELISA before (day 0) and after 7 and 14 days post Hsp65 inoculation. *p < 0.05 and **p < 0.01 (Two-way ANOVA, Bonferroni multiple comparisons post-test).

Since the antibody production against heat shock proteins are involved in autoimmune processes, we measured the anti-DNA and anti-Hsp65 IgG1 and IgG2a isotypes production after WT and point-mutated Hsp65 inoculation. In aged H_III_ female mice, WT and K^409^A inoculation caused a 4.5-fold increase in anti-Hsp65 IgG1 production (*p <* 0.01) (Figure 3A), compared to pre-immune animals (zero days), at fourteen and seven days post-inoculation, respectively. Despite the reduction observed in IgG2a anti-Hsp65 (Figure 3A) and IgG1 anti-DNA (Figure 3B) observed in aged H_III_ female mice, the non-inoculated group (control) also showed this decrease, possibly indicating an environmental interference non-related with the protein injection. No differences in IgG2a anti-DNA production were observed.

Antibody production kinetics analysis of aged H_III_ female mice shows an increase of the IgG1 anti-Hsp65 (*p <* 0.01) in the WT-injected group, starting at 2.8 log_2_ and reaching 5.4 log_2_ on day fourteen, and 3.3 log_2_ to 6 log_2_ in K^409^A group (*p <* 0.05) on 7^th^ day post-immunization when compared to pre-immune serum (data not shown).

**Figure 4:**
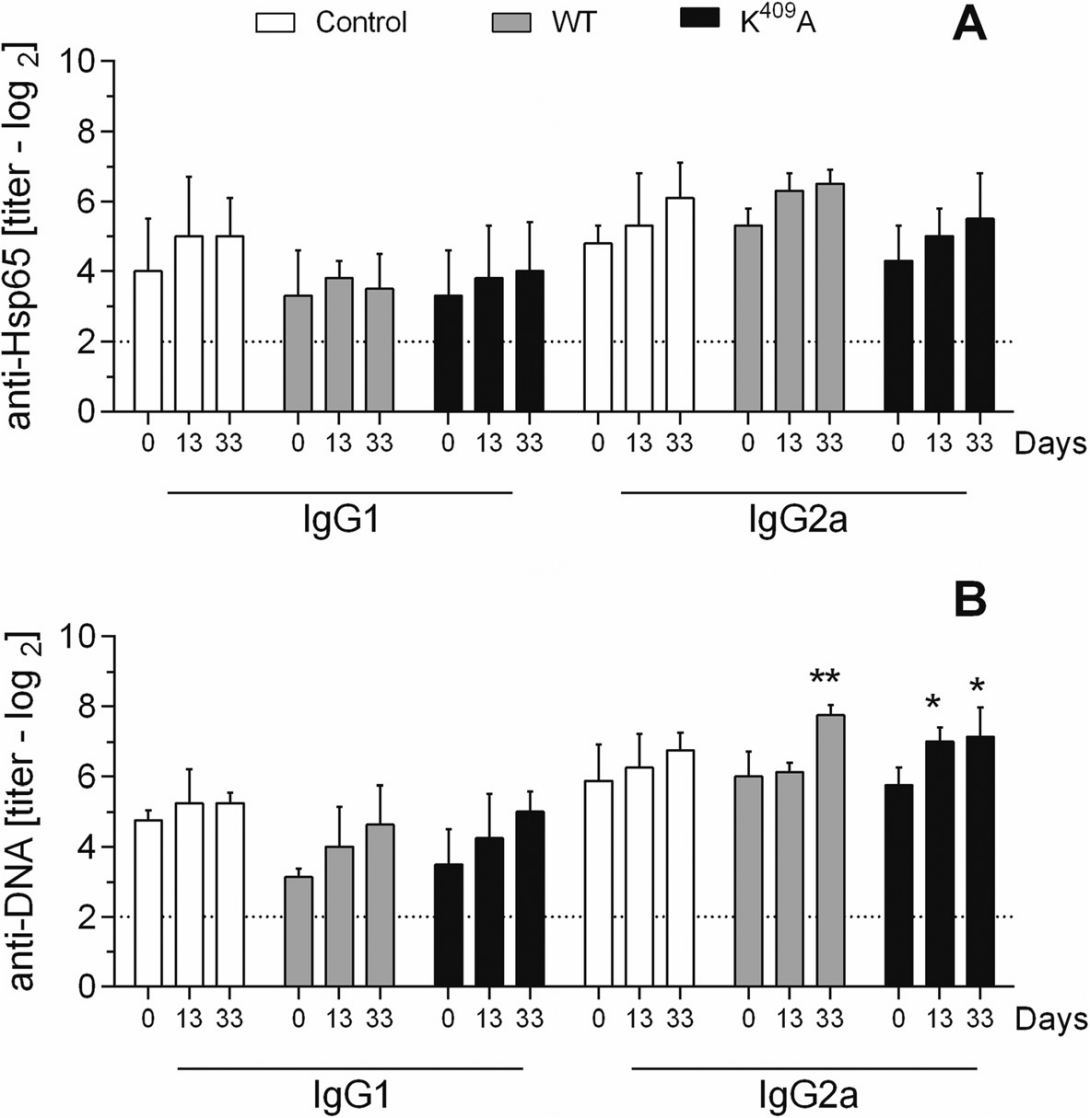
**Anti-Hsp65 and anti-DNA antibodies production in adult H**_**III **_**female mice.** Anti-Hsp65 IgG1 and IgG2a **(A)**, and anti-DNA IgG1 and IgG2a **(B)** isotype production in the serum of adult H_III_ female mice (4 animals/group) inoculated with WT, K^409^A Hsp65, or PBS (control). Antibody titers were determined by ELISA before (day 0) and after 13 and 33 days post Hsp65 inoculation. *p < 0.05 and **p < 0.01 (Two-way ANOVA, Bonferroni multiple comparisons post-test).

In adult H_III_ female mice, the administration of both WT and K^409^A Hsp65 molecules did not promote alterations in the anti-Hsp65 antibody production kinetics between all experimental groups until 33 days post-inoculation (Figure 4A). However, the anti-DNA titer was increased at 33^rd^ days in WT (*p <* 0.01) and K^409^A (*p <* 0.05) groups (Figure 4B). Regarding aged H_III_ male mice, the isotypes titration showed only increased IgG1 anti-Hsp65 isotype production compared to pre-immune groups at the 14^th^ day after the K^409^A Hsp65 injection (data not shown).

**Figure 5:**
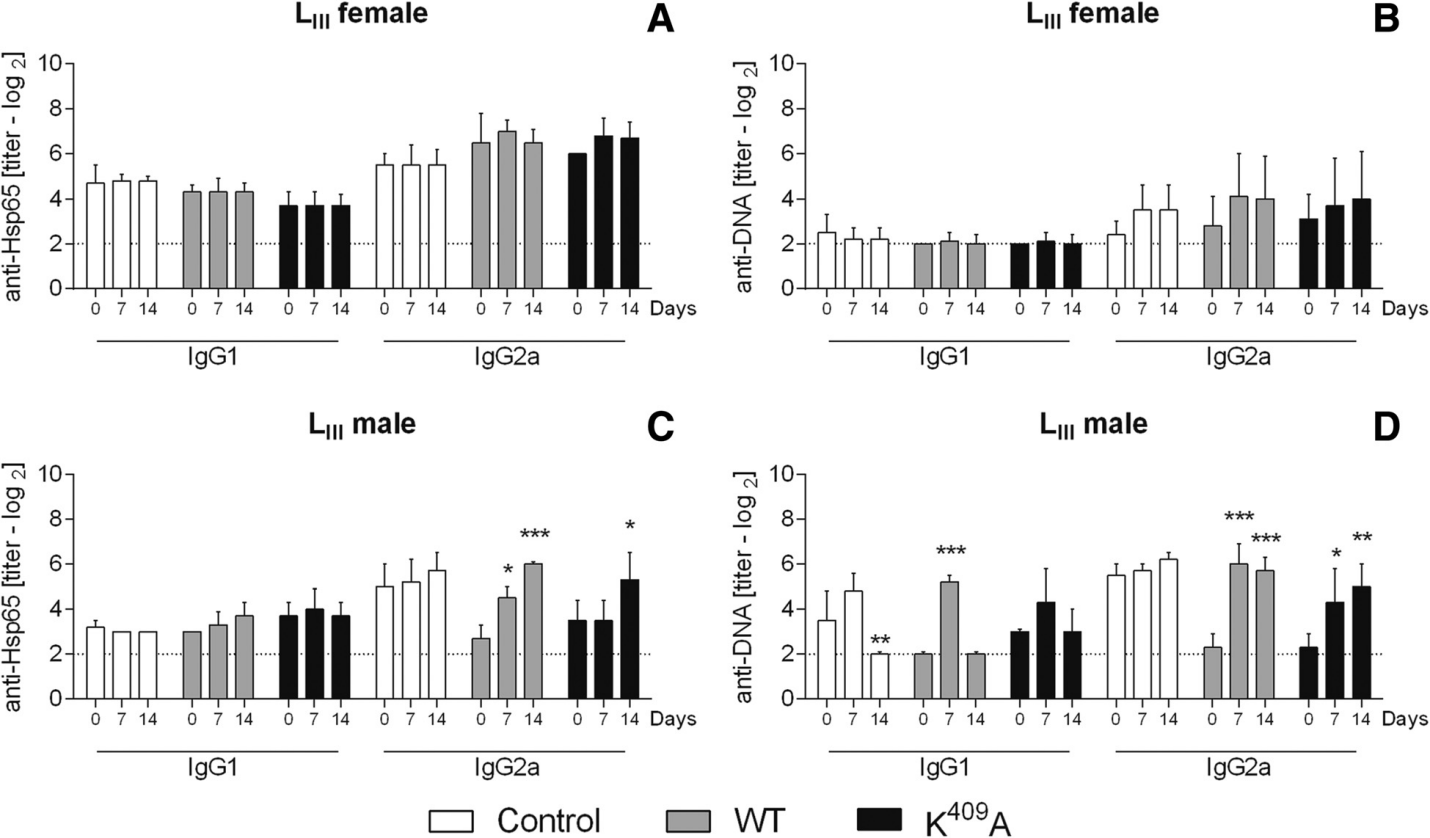
**Anti-Hsp65 and anti-DNA antibodies production in aged L**_**III **_**mice.** Anti-Hsp65 IgG1 and IgG2a **(A and C)** and anti-DNA IgG1 and IgG2a **(B and D)** isotype production in the serum of aged L_III_ mice (4–8 animals/group, males and females) inoculated with WT, K^409^A Hsp65, or PBS (control). Antibody titers were determined by ELISA before (day 0) and after 7 and 14 days post-WT or -K^409^A Hsp65 inoculation. *p < 0.05, **p < 0.01 and ***p < 0.001 (Two-way ANOVA, Bonferroni multiple comparisons post-test).

After the observation of the increased survival of aged L_III_ female mice injected with WT Hsp65, we investigated the effects of those molecules over the antibody production in low antibody responder mice. No differences were observed in IgG1 (titers range from 3.7 ± 0.8 to 4.2 ± 0.5 log_2_) and IgG2a (from 5.5 ± 0.8 to 7.0 ± 1.0 log_2_) anti-Hsp65 or IgG1 (from 2.0 ± 0.4 log_2_ to 2.5 ± 0.8 log_2_) and IgG2a (from 2.4 ± 0.5 log_2_ to 4.0 ± 1.9 log_2_) anti-DNA production in aged L_III_ female mice after WT or K^409^A Hsp65 inoculation (Figure 5A and B). On the other hand, L_III_ male mice showed elevated levels of IgG2a anti-Hsp65 after 14 days subsequently the WT (*p <* 0.001) or K^409^A (*p <* 0.05) injection (Figure 5C), whereas the anti-DNA data displayed mainly an increase in IgG2a anti-Hsp65 after WT (*p <* 0.001) and K^409^A (*p <* 0.01) inoculation (Figure 5D).

## Discussion

Immunological functions may change with aging and lead to a deficient immune response to several infections and vaccines, predisposing the individual to chronic-degenerative processes by the decline of self-tolerance maintenance and loss of tissue integrity, directing to crypt antigens release, amplified bystander activation and molecular mimicry events [2,41,42]. Based on the immune alterations observed in the aging process, the reports of higher incidence of chronic-degenerative processes in elders [14,43] and the deleterious effect of *M. leprae* Hsp65 administration on murine lupus and autoimmune uveitis [35,38], we evaluated the interference of WT Hsp65 administration on survival time and correlation with antibody production in H_III_ and L_III_ mice. The animals from Selection III are a well-established model to understand the humoral immune response and its influences over the susceptibility to infections [44], autoimmunity [45] and tumorigenesis [46]; they also differ in the response to antigens not related to those used for the selection procedure [47] and present different susceptibility to autoimmune disease [48]. Moreover, H_III_ and L_III_ mice are used to verify the influence of genetic alterations over longevity, as demonstrated by the differences between the survival of distinct genetic bidirectional selections: Selection I and II presents different survival between lines and gender and Selection III shows similar mean survival time regardless of sex or linage (H_III_: 611 ± 153 days, L_III_: 622 ± 166 days) [49,50].

Native Hsp65 effects on survival of aged and adults H_III_ female mice indicate the immunological interference of the Hsp65 molecule in this model. It reduced the survival of H_III_ female mice, mainly in young aged (270-days old), with first death occurring 18 days after WT administration, whereas for adults H_III_ female mice (120-days old) it occurred 247 days post-injection, a period 14-times greater than the aged mice. In opposition, WT-inoculated aged L_III_ female mice presented higher MST (615 ± 46 days, *p <* 0.01) compared to mutant and control groups (442 ± 97 and 441 ± 72 days, respectively). Despite the differences of the maximal survival time in control groups (Additional file 1), we can take these data in account as the maximal survival obtained was within to those previously observed [50], with 501 days in aged L_III_ female and 759 days in aged L_III_ male mice. The maximal survival of control group of H_III_ female (763 and 765 days) or L_III_ male (759 days) showed a significant difference because some mice died with extreme lifespan. Despite the low number of animals used (restricted by the ethics committee) the results demonstrated that the *M. leprae* Hsp65 injection could alter the survival, reducing MST in H_III_ and augmenting in L_III_ female mice.

Considering that L_III_ line shows an 18-fold reduction in the antibody production and low T cell proliferation [51], it is possible to consider that the humoral response of L_III_ mice against Hsp65 is reduced, resulting in an easy management by the system to return to homeostasis. The same could be true for other mice lines with lower antibody response compared to H_III_ mice, like Swiss albino mice (foundation of Selection III lines) [47]. Contrary, the high responder rate of the immunological system in H_III_ female mice after WT injection, despite the antibody production rate, could disrupt the homeostasis and lead to a reduced survival. Homeostasis imbalance due to aging process in association with the interference of Hsp65 inoculation could explain the higher decrease in survival of H_III_ aged female. In spite of our animal model do not spontaneously develop autoimmune processes, the reduced survival time of H_III_ female mice matches to the experiments involving lupus-prone mice [35] and models of experimental autoimmune uveitis in mice [38], and reassure the involvement of Hsp in chronic-degenerative processes. The control expression and the rupture of Hsp65 balance in SLE development were ascertained through the approach of inductive disequilibrium of physiological and immune states by homologous Hsp [52] and the same could be true for the current study.

Since High and Low mice lines differ, respectively, to high or low antibody responses, the anti-DNA and anti-Hsp65 IgG isotypes production were analyzed after WT or K^409^A Hsp65 inoculation. H_III_ and L_III_ mice, both genders, presented slightly higher production of IgG2a anti-Hsp65 and anti-DNA related to the IgG1 isotype. This balance towards a Th1 response may indicate a natural pro-inflammatory status in these strains which is confirmed by the relative easy way to induce adjuvant arthritis in H_III_ mice [53]. The time-course analyses of immunoglobulin production did not show significant intragroup differences, which might be related to the absence of a strong specific response to these proteins demonstrated by the low antibody titers even 30 days after the protein inoculation and because this is not an immunization process. Compared to pre-immune mice, aged H_III_ females presented an increase of anti-Hsp65 IgG1 in the group treated with WT or K^409^A Hsp65 and a non-related reduction of IgG2a. In murines, the IgG1 and IgG2a functions are dependent on the cells activation threshold determined by the affinity of antibodies and the expression of inhibitors/activators receptors [54]. The amplification of anti-Hsp65 IgG1 antibody (approximately 4-times) should be related with a switch to a Th2 response, previously observed in the immunosenescence process [2]. It also could correlate to the precocious death of aged H_III_ female mice, since the augment of Th2 cytokines, despite its regulatory effect, are involved in some diseases like asthma, allergies and autoimmunities [55,56].

Confirmed the *M. leprae* Hsp65 effect on reduced survival in aged H_III_ female mice, it was evaluated whether the anti-DNA and anti-Hsp65 antibody production were age-dependent by comparing the IgG isotypes with adults H_III_ female mice. They did not present intergroup changes in kinetics of anti-DNA or anti-Hsp65 antibodies production, but adult H_III_ female mice showed increase in IgG2a titers after Hsp65 molecules injection. The IgG2a isotype titers are remarkably lower in adult mice compared to aged ones as an indicative of a better balance of Th1/Th2 response and maintaining homeostasis of the immune system, as demonstrated by the 13-times later death in adult H_III_ females after WT inoculation compared to old mice.

This dominating effect observed in the survival time of aged mice emphasizes the involvement of the Hsp65 molecule in aging processes. The effect of native molecule was gender-specific, as demonstrated by the unaltered MST in aged male mice (H_III_ and L_III_) inoculated with both proteins, and potentially related with the differential regulation of the immunological system by sexual hormones [57], as the dimorphism between gender is positively linked with different susceptibility for infections, autoimmune diseases and tumor incidence [40,58]. Sexual hormones (mostly estrogen but also progesterone and testosterone) affect immune cells quantitatively and qualitatively and impact on cytokine production [59]. Females have higher plasma concentrations of immunoglobulin, increased number and strong activation of CD4^+^ T cells, elevated levels of Th1 cytokines (IgG2a production) and stronger primary and secondary antibody response [60,61]. Indeed, the higher incidence of SLE in females reflects the gender dimorphism [62,63].

It should also be considered the animal model used to test the relationship of Hsp and aging. The opposite phenotypes of antibody production in H_III_ and L_III_ mice include immune response to a wide range of antigens [47]. The F_0_ - foundation population - of Selection III mice are genetically heterogeneous: the phenotypic variance (V_P_) is due to the sum of the genetic variance (V_G_), and the environmental variance (V_E_) emerges by all the causes of variability resulting from the environment. The bi-directional selection resulted in a progressive increase (H_III_ mice) or a decrease (L_III_ mice) in mean antibody response, followed by the decline of V_G_ in both lines [47,64]. Therefore, the alterations provoked by WT or K^409^A Hsp65 administration (the environmental feature applied during the experiments) provide the variance in our experimental model. After WT inoculation, a great reduction in variance value was observed in aged female H_III_ (V_E_ = 635 versus V_E_ = 15293 of the control group) than L_III_ mice (V_E_ = 2180 versus V_E_ controls = 5328), showing the impact of this molecule administration over the potential of death phenotype (MST = 308 ± 25 in aged H_III_ and MST = 615 ± 46 in aged L_III_ female mice). We cannot exclude the interference of others genetic factors occurred during the selective breeding, and a gender-effect that may affect the response in H_III_ and L_III_ mice. Since it was proposed that the presence of anti-Hsp60 autoantibodies, innate risk factor of atherosclerosis in adulthood, may be an inherited trait, we are conducting studies about the effect of Hsp65 in (H_III_xL_III_)F_1_ hybrids to clarify the genetic influence of this susceptibility [65].

Mechanisms underlying the distinct effects of the native Hsp65 on survival of H_III_ and L_III_ mice, and the comparison between them and mutant injected individuals are under evaluation. Preliminary histopathological analysis with some control- and WT-injected H_III_ female mice (3 animals/group) indicates that the WT Hsp65 inoculation results in a widespread chronic hepatitis, spleen hyperplasia, and, unlike L_III_ female, higher degree of nephrosis and chronic nephritis with inflammatory infiltration of plasma cells, macrophages and lymphocytes (data not shown), characteristics also present in human lupus nephritis [66]. Studies of immune cells alterations in spleen and blood samples of aged H_III_ female mice injected with *M. leprae* Hsp65 are in progress. The initial results shows increased splenic B cells percentage, amplified expression of CD45RA and CD154 in CD4^+^ T cells, reflecting on the augmented anti-Hsp65 IgG1 isotype production observed here, and amplified surface expression of CD11b and CD11c in blood monocytes.

The adaptive management of biological systems according to environmental changes is essential for the organism survival and Hsp molecules can interfere with immune phenotypes submitted to independent polygenic control. The aging process presents an impaired cellular homeostasis and the Hsp presentation by antigen presenting cells may be diminished, being responsible for the decline in immunoregulation through the recognizing of self Hsp [67]. On the other hand, an amplified expression of stress proteins and his antigenic determinants can evolve to a pathogenic or regulation of chronic-degenerative processes [68-70]. Taking together, these facts explain the pleiotropic effects of Hsp65 on biological systems and its wide actions over different cell types and production of other molecules. Based on pleiotropy, the *Theory of Aging* proposed by George C. Williams suggests that some genes responsible for increased fitness in young fertile individuals may contribute to the reduction of such capacity in later life [71]. Then, it is conceivable that selection for high antibody production genes, essential for the immune protection through the life of an individual, can be one of the factors that allows Hsp65 act on the immune or physiological imbalance later in life. As previously reported by our group, the WT Hsp65 passive administration affects the endogenous balance by increase the entropy of the individual system; the linear equation proposed (y = a + Δ*i*) shows that the immunological history (y) is unique, irreversible and cumulative [35]. In this study, the animal model employed is not naturally predisposed to autoimmunity, so “a” should include the potential advent of chronic-degenerative processes in aging and “*i*” consists by the sum of the environmental factors that modulate the entropy: age, gender, antibody production rate, and possible cellular and molecular alterations established in Selection III after Hsp65 administration. All these elements interfere in how Hsp65 interact with the immune system; consequently, the greater their influence, greater the entropic energy, hindering the recovery of homeostasis and contributing to the deaths of H_III_ female mice.

Despite the absence of strikingly differences in antibody production in our experiments, perhaps the 2-fold higher antibodies production in H_III_ females compared to males [47], associated with the senescent immune system and influenced by hormones, are sufficient to induce frailty after WT administration. In parallel, the high antibodies production rate in H_III_, besides increasing the system entropy, can result in reduced antibody affinity for the protein, facilitating its subsequent binding to self-antigens. In case of imbalance due to the overstimulation by stress or inflammation, autoimmune diseases may emerge or aggravate [35,52]. The opposed occurs in L_III_ females, which presented increased survival when injected with the native molecule, suggesting that the low immunoglobulin production may favor the control of immune system overstimulation.

We do not observed any signs of disease development during the survival time assay and this be correlated with the incapacity of the mycobacterial Hsp65 alone to induce, in some cases, autoimmunity. In an experiment of arthritis induction by Complete Freund’s adjuvant (CFA) replaced with the whole mycobacterium [72], the intradermal injection induced arthritic lesions at the same degree as CFA in ankle joints, with the production of anti-DNA and anti-Hsp65 in rats. Thus, the not remarkable increase of anti-Hsp65 antibodies presented by H_III_ and L_III_ mice may be responsible for the absence of disease.

More than a phenotypic effect by the antibody production against WT Hsp65, the extended pleiotropic effect of this protein over the immune system results in strengthening of naturally established disorders in aged H_III_ female mice who possibly present a natural higher degree of entropy. In addition, the age-remodeled immune system already shows a major entropy level and the injection of *M. leprae* WT Hsp65 in females reinforce an imbalance that does not resemble the young individuals, originating disorganizations and irreversible processes leading to death.

## Conclusions

Here we verified in an aging mice model the role of *M. leprae* Hsp65 in the aggravation of phenotypes, as observed in SLE and experimental autoimmune uveitis, and outlined its interference mainly in aged H_III_ female inducing precocious death. We assume that this effect is associated to the aging process and related to gender-effect instead of the amount of antibody produced in these mice lines. Studies of cellular and cytokines alterations after the Hsp65 administration in Selection III mice and its (H_III_xL_III_)F_1_ hybrid mice are in progress to elucidate the mechanisms by which this heat shock protein and its responses act in the immune system of aged individuals.

## Methods

### Expression of the recombinants *M. leprae* Hsp65 in *Escherichia coli* and purification

Clone pIL161, containing the coding sequence of the *M. leprae* WT Hsp65 and its point-mutated form K^409^A [36] were amplified in *E. coli* DH5a cells. Expression and purification of the recombinant Hsp65 WT and K^409^A was described elsewhere [35].

### Animals

The genetically heterogeneous selected mice for High (H_III_) or Low (L_III_) antibody production were bred in the animal facility of the Immunogenetic Laboratory and maintained at the animal facility of the Immunochemistry Laboratory, both in Butantan Institute. They were housed in groups of four to five in plastic cages filled with hardwood bedding, provided with water and rodent chow *ad libitum*, in a room with 12-h light/dark cycle, controlled pressure, humidity and temperature (24 ± 2°C). All procedures are in agreement to the International Animal Welfare Recommendations [73] and the experiments are in conformity with the Ethical Principles in Animal Research, adopted by the Brazilian College of Animal Experimentation, and was approved by the Ethical Committee for Animal Research of Butantan Institute (CEUAIB #475/08).

### Administration of the WT and K^409^A Hsp65 molecules

Male and female H_III_ and L_III_ mice at the age of 120- or 270-days old (named here “adult” or “aged” mice, respectively) were inoculated intraperitonially with a single dose of 2.5 μg of WT or K^409^A Hsp65 of *M. leprae* in 0.2 mL of phosphate buffer saline pH 7.4 (PBS); as controls, mice were injected with 0.2 mL PBS. In this study it is important to highlight that the periodically bleedings were performed at different time points in aged and adult female mice; from previously observations that aged H_III_ female animals death started 18 days after the WT Hsp65 administration (bleedings occurred at seven and at fourteen days post proteins administration). Differently, adult H_III_ female individuals, which presented an extended survival time after the WT Hsp65 inoculation, were bled after fourteen and thirty-three days after the proteins injection; this longer interval was used to avoid external stress stimuli that could influence the experiment. The serum samples were stored at -20°C until antibody titration. Each animal was observed until death for the designing of the longevity curve and examined for clinical signs that include development of ascites and lethargy.

### Titration of anti-DNA and anti-Hsp65 antibodies

Levels of anti-DNA and anti-Hsp65 IgG1 and IgG2a isotypes titers were set by indirect ELISA as describe before [35] and expressed as log_2_ of the reciprocal serum dilution giving an absorbance value of 20% of the saturation level.

### Statistical analyses

All statistical analyses were performed with GraphPad Prism 5.0 software. The antibody dosages are expressed as mean ± SD. Two-way ANOVA with Bonferroni multiple comparisons post-test were used to evaluate the antibody production between mice from control, WT and K^409^A groups. Kaplan-Meier plot for mean survival time (MST) was analyzed by log-rank test (Mantel-Cox) comparing the MST with age, dose or administration period of WT or K^409^A rHsp65. For all data, minimum statistical significance was set at *p*<0.05.

## Competing interests

The authors declare that they have no competing interests.

## Authors’ contributions

EJB, EBM, VB and OAS participated in the design of the study and writing of the manuscript. EJB performed the assays and analyzed the data. MDF and NS provided the mice used in all experiments and participated in the design of the experiments. VB assisted the discussion of results and writing of the manuscript. All authors read and approved the final manuscript.

## Supplementary Material

Additional file 1 **Percent survival of control groups in Selection III mice.** H_III_ and L_III_ from control group, used in survival analysis, were compared. The adults (270-days old) and young aged (120-days old) female H_III_ mice where analyzed together. Those mice received PBS (200 μL/animal) at 120- or 270-days as previously described. Statistical analysis (log rank test (Mantel-Cox)): ^a^*p <* 0.05 H_III_ male versus H_III_ female; ^b^*p <* 0.05 H_III_ male versus L_III_ male; ^c^*p <* 0.05 H_III_ female versus L_III_ female and ^d^*p <* 0.01 L_III_ female versus L_III_ male. Click here for file
