# Supplementary figures and images for: Mycobacterium leprae Hsp65 administration reduces the lifespan of aged high antibody producer mice
Source: Immun Ageing. 2014 Mar 26;11:6. doi: 10.1186/1742-4933-11-6 (PMC3986931; doi:10.1186/1742-4933-11-6)

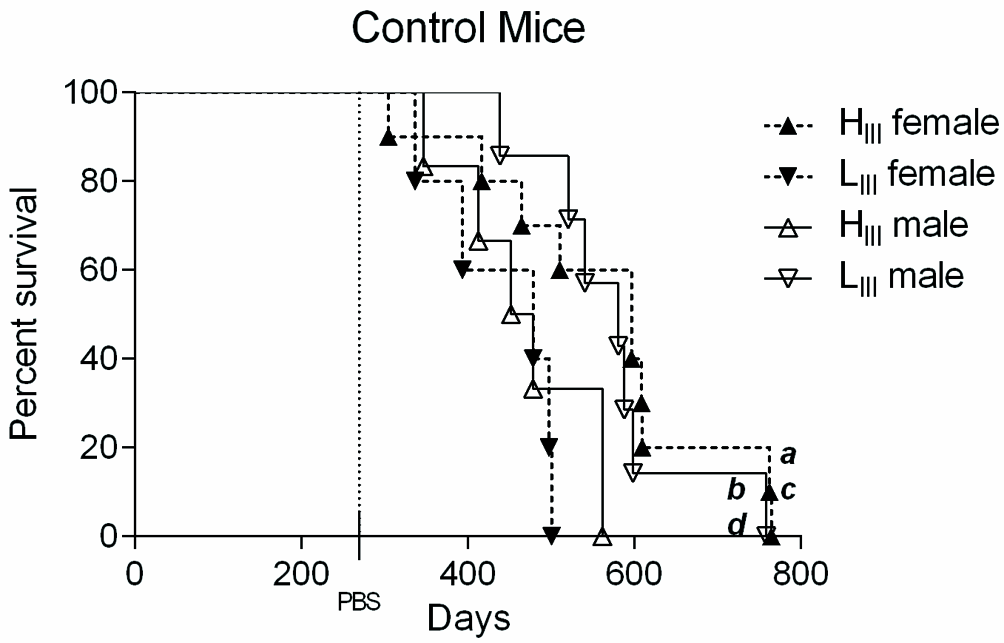

Supplement: Additional file 1 — Percent survival of control groups in Selection III mice. HIII and LIII from control group, used in survival analysis, were compared. The adults (270-days old) and young aged (120-days old) female HIII mice where analyzed together. Those mice received PBS (200 μL/animal) at 120- or 270-days as previously described. Statistical analysis (log rank test (Mantel-Cox)): ap < 0.05 HIII male versus HIII female; bp < 0.05 HIII male versus LIII male; cp < 0.05 HIII female versus LIII female and dp < 0.01 LIII female versus LIII male. [file 1742-4933-11-6-S1.tiff]
